# ^1^H-NMR Profiling and Carbon Isotope Discrimination as Tools for the Comparative Assessment of Walnut (*Juglans regia* L.) Cultivars with Various Geographical and Genetic Origins—A Preliminary Study

**DOI:** 10.3390/molecules24071378

**Published:** 2019-04-08

**Authors:** Raluca Popescu, Roxana Elena Ionete, Oana Romina Botoran, Diana Costinel, Felicia Bucura, Elisabeta Irina Geana, Yazan Falah Jadee ’Alabedallat, Mihai Botu

**Affiliations:** 1 National Research and Development Institute for Cryogenics and Isotopic Technologies-ICSI Rm. Valcea, ICSI Analytics Group, 4 Uzinei Street, RO-240050 Râmnicu Vâlcea, Romania; raluca.popescu@icsi.ro (R.P.); oana.dinca@icsi.ro (O.R.B.); diana.costinel@icsi.ro (D.C.); felicia.bucura@icsi.ro (F.B.); irina.geana@icsi.ro (E.I.G.); 2 University of Craiova, Faculty of Horticulture, Department of Horticulture and Food Science, 13 Al. I. Cuza Street, RO-200585 Craiova, Romania; yazan.fja@gmail.com (Y.F.J.’A.); btmihai2@yahoo.com (M.B.); 3 University of Craiova, Fruit Growing Research Station (SCDP) Vâlcea, 464 Calea lui Traian Street, RO-240273 Râmnicu Vâlcea, Romania

**Keywords:** *δ*^13^C-IRMS, fatty acids composition, ^1^H-NMR, walnut varieties

## Abstract

The aim of the study was to investigate the differences between walnut genotypes of various geographical and genetic origins grown under the same or different environmental conditions. The biological material analyzed consisted in walnut kernels of 34 cultivars, nine advanced selections, and six hybrids harvested in 2015 and 2016, summing up to a total of 64 samples. The walnuts, walnut oil, and residue were characterized in respect to their chemical (proximate composition—fat, protein, nutritional value, fatty acids profile by ^1^H-NMR) and carbon-13 isotopic composition. The data was used to statistically discriminate the cultivars according to composition, geographical area of origin, and year of harvest, comparing the Romanian cultivars, selections, and hybrids with the internationally available ones.

## 1. Introduction

Rich in omega-3 fats, proteins, and with a higher number of antioxidants than most other foods, walnuts are recognized as an important component of a healthy diet. The walnut kernels’ composition contains between 52 and 77% fats, 12 ÷ 25% protein, 5 ÷ 24% carbohydrates, quinones, tannins, minerals, and vitamins (A, B1, B2, P, and C), that may significantly differ with the genotype, growth location, and environmental conditions [1]. Compared to other types of nuts, they are rich in terms of polyunsaturated fatty acids (PUFA), such as omega-6 and omega-3 [2], the major fatty acids found in walnut being linoleic (C18:2), oleic (C18:1), α-linolenic (C18:3), palmitic (C16:0), and stearic acids (C18:0) [3,4]. 

Consumers are becoming increasingly aware of food quality and authenticity of the nutritional composition and health-promoting components. In this regard, walnuts have generated considerable interest since it was confirmed by various survey studies [5,6,7] that their consumption can lower the cholesterol, improve the arterial function (by decreasing total and LDL-cholesterol, and increasing HDL-cholesterol), reduce inflammation, and decrease the likelihood of diabetes and neurological diseases. The beneficial components for health found in walnuts are the polyunsaturated fatty acids, phytosterols, proteins, biogenic amines (melatonin and serotonin), dietary fiber, folate, tannins, polyphenols, and minerals (magnesium, potassium, calcium, etc.) [1,2].

Covering a wide and diverse expanse of land in Central Asia [8], the walnut has spread in China, India, Europe, and America, and is nowadays cultivated in almost all countries with temperate climates. The walnut belongs to the Juglandaceae family that contains six genera for the temperate region, with the most important two being *Juglans* and *Pterocarya*. The *Juglans* genus contains 12 species, from which the high feasibility in obtaining new cultivars and parent stock are the *Juglans regia* L. and *Juglans nigra* [9]. Persian walnut (*Juglans regia* L.) is mostly cultivated in Asia (with China as the top producer), the U.S., and Europe. Of the European countries, Romania is in the top three for the yield/hectare and in the top 10 for the production and export [10], cultivating different varieties chosen according to their high yield, disease resistance, or product quality.

Evaluating and certifying the identity/origin and quality of walnuts, and their byproduct, the oil, is a challenge for the food industry, regulatory bodies, and consumers. There are various developed methods to differentiate vegetable oils, including nuts oil, and the most recent are the nuclear magnetic resonance-based metabolomics [11,12] and stable isotopes investigation [13,14,15], along with chemometrics [16]. Generally, the studies on walnuts investigated the major and minor compounds, such as fatty acids, sterols, polyphenols, volatiles, and minerals [17,18], or assessed the oxidative stability, antioxidant, and antimicrobial activity [1,19,20] to determine the variations given by the location, environmental conditions, cultivars, and technological processing [21,22,23]. Although the main purpose of isotopic studies is to identify the botanical and geographical origins of different food products, only a few were applied to walnuts and walnut oil [24,25,26], but lacked reference to the variation given by the cultivar/genotype, geographical origin, or year of production (environmental conditions—temperature, rainfall, and light).

As a consequence, the aim of the present work was to investigate the differences between walnut genotypes of various geographical and genetic origins grown under the same or different environmental conditions. The analyzed biological material consisted in walnut kernels of 34 cultivars, nine advanced selections, and six hybrids from the breeding program, harvested in 2015 and 2016. The walnuts, walnut oil, and residue were characterized in terms of chemical proximate composition (fats and protein content, nutritional value, fatty acids profile by ^1^H-NMR) and carbon-13 isotopic composition. The data were used to statistically discriminate the cultivars according to the composition, geographical area of origin and year of harvest, comparing the Romanian cultivars, selections, and hybrids, with the internationally available ones. 

## 2. Results and Discussion

### 2.1. Geographical and Year of Harvest Discrimination

Chemical composition of the kernel, oil, and residue for the walnut samples is highlighted in Table 1. The samples are grouped according to the year of harvest and geographical origin of the walnut cultivar, with an emphasis on the Romanian cultivars and the selections and hybrids developed at the SCDP Râmnicu Vâlcea (Romania).

The walnut kernels contained mainly fats (69.0%), with a mean concentration of crude protein of 20.3% and a 10.7% content of carbohydrates, resulting in a 700 kcal/100 g kernel energy. The extracted oil, with a mean of 874 kcal energy, was composed mainly of linolenic acid (as sum of ω-6 fatty acids)—55.6% molar, followed by 22.7% monounsaturated fatty acids (mainly oleic), 11.1% linoleic acid (as sum of ω-3 fatty acids), and 10.6% saturated fatty acids, giving a mean unsaturation degree (IV–iodine value) of 139. 

The fatty acids composition allowed the estimation of the different nutritional fractions: PUFA—polyunsaturated fatty acids, UFA/SFA—unsaturated fatty acids/ saturated fatty acids, PUFA/SFA—polyunsaturated fatty acids/saturated fatty acids. The results as a general composition for the walnuts and walnuts oil and for specific cultivars were in agreement with other published data [1,17,20,22,23,27].

Considering the walnuts grown at the Fruit Growing Research Station (SCDP) Vâlcea, the Romanian selections and hybrids tend to present the extreme values for this set of samples. The Romanian hybrids registered high protein content (26.0%) and low fats (66.5%) for the kernel and oil, with a low composition in oleic acid (18.8% molar), and high linoleic acid (59.2% molar), that led to increased values of PUFA (70.4 % molar) and IV (142) compared to the other groups of walnuts.

The Romanian selections were also found to have a low-fat content (66.3%), with a higher oleic concentration (24.7% molar) and lower linoleic (54.8% molar) and linolenic (10.1% molar) concentrations, resulting in low PUFA. The international and Romanian cultivars had similar compositions, the values being intermediate between the Romanian hybrids and the Romanian selections, with the exception of the higher fatty content for the French cultivars (mean of 73.6%) and higher PUFA/SFA and UFA/SFA ratios for the USA cultivars.

For harvest year, discriminations were considered only among the cultivars from the Fruit Growing Research Station (SCDP) Vâlcea sampled both in 2015 and 2016, namely 15 cultivars, from which 10 were international and five Romanian ones. The classification of the samples was 100% correct, with the *δ*^13^C of the residue (which is the carbon-13 fingerprint of the proteins and carbohydrates), protein content of the kernel and polyunsaturated fatty acids concentration, and the iodine value as the most important factors for discrimination. The walnuts from 2015 had a lower content of fats with a higher degree of unsaturation than the ones harvested in 2016, together with a higher content of proteins. Also, since in 2015 there were higher mean temperatures and precipitation in the maturation period (July–September) than in 2016 [28], a possible explanation could be drawn that increased temperatures and precipitation are conductive to the formation of proteins in the kernels and lower quantities of fat with higher concentrations of polyunsaturated fatty acids.

**Figure 1:**
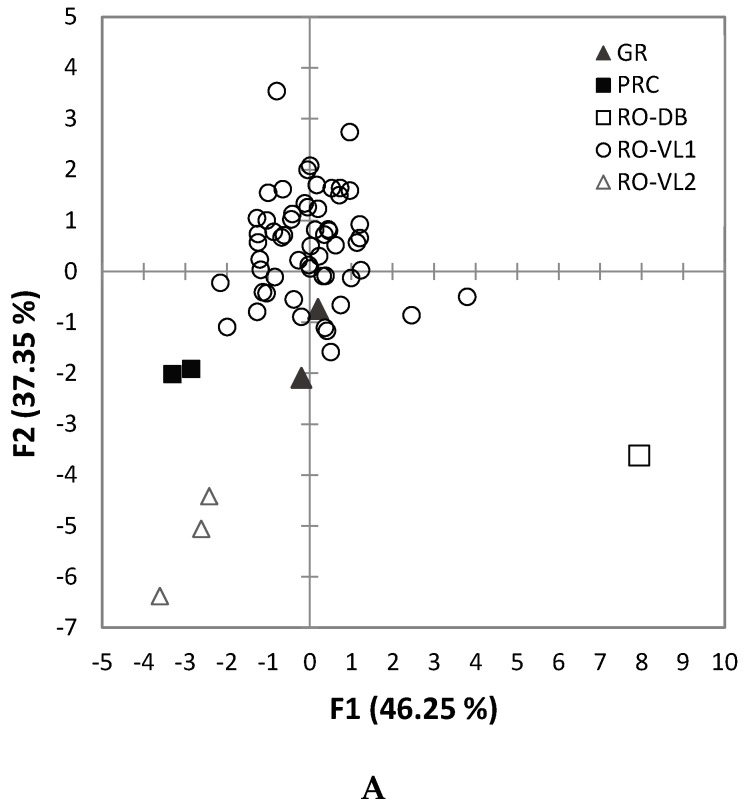

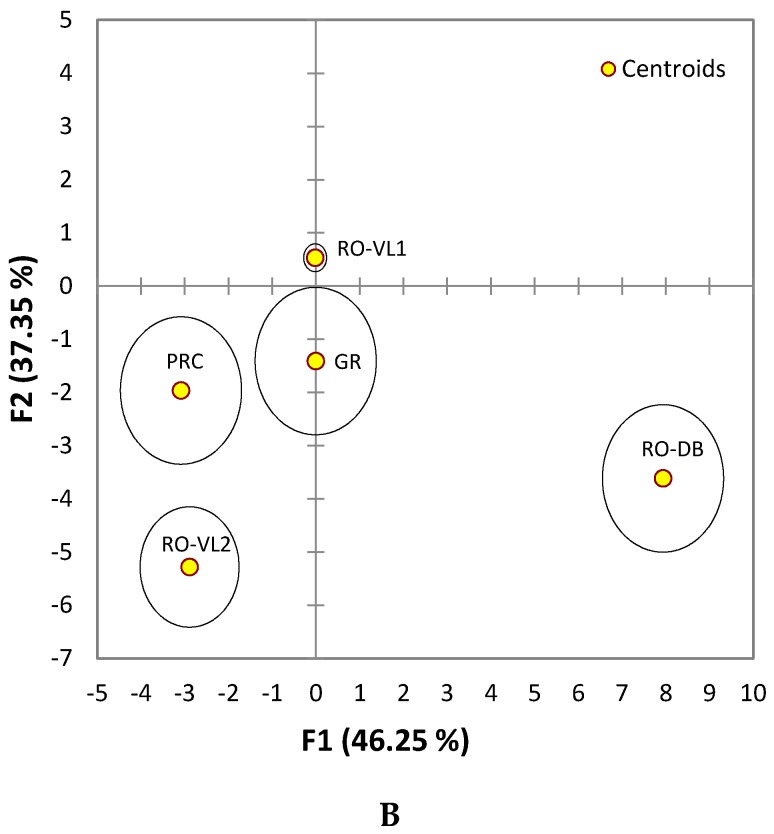
Discriminant analysis of walnuts according to the geographical origin of the walnut samples: (**A**) samples distribution by groups; (**B**) group centroids as mean discriminant scores for each group in the dependent variable for each of the discriminant functions.

Discriminant analysis of walnuts grown in different locations is presented in Figure 1. For all samples, the first two most important factors summed 83.59% of the variance (F1 = 46.25% and F2 = 37.35%), with 100% correct classification of the samples. 

The most important parameters for the geographical discrimination of walnuts were the *δ*^13^C of the kernel, oil, and residue, the SFA, oleic concentrations, together with the indices IV, UFA/SFA, and PUFA/SFA. Since the number of samples grown in different locations than RO-VL1 was low, no general conclusions could be drawn. As a general observation, the two samples from China presented high oleic composition (38.5 and, respectively, 31.2% molar) compared with the other cultivars, resulting in a low IV of 129.

Other distinct compositions were observed for the walnut with red kernels (RO-DB), which had high fatty content (79.6%) with higher SFA (13.4% molar), and for the Romanian selections RO-VL2 (*n* = 3) which had low oleic and high linoleic/linolenic concentrations (mean of 60.5% and, respectively, 12.4% molar) resulting in a high IV (144).

### 2.2. Stable Carbon Isotopic Composition

The carbon stable isotopes are important for biological, cultivar and climatic differentiation [24,29,30]. Variation of *δ*^13^C values between different plants and plant products may be attributed, besides the metabolic pathway, to geographical origin, environmental factors (temperature, humidity, rainfall, light exposure, water availability), cultivar, growth conditions and harvest time [26].

The variation in carbon-13 abundance in food is the result of isotopic fractionation that takes place in plants [25]. During the photosynthesis, the light (^12^C) and heavier (^13^C) isotopes of carbon will be discriminated, the processes being reflected in the isotopic compositions of the plant components. Carbon dioxide-fixing pathways in plants are the C3 (Calvin cycle), C4 (Hatch-Stack cycle), and CAM (Crassulacean acid metabolism), with the mention that all trees use the C3 metabolism. Since walnut is a C3 plant, the isotopic fingerprint of the kernel and oil should be in the range −34‰ to −22‰ [24]. Studies done on walnut and walnut oil samples produced in France and China, reported values of −28.67 ± 0.25‰ [25] and, respectively, −27.5‰ [24,25,26] for the *δ*^13^C.

In this study, the range of variation for the *δ*^13^C of the kernel was of 7.6 delta units (between −29.9 and −22.3‰), while for the oil and residue was about six delta units, between −30.1 and −23.9‰ and, respectively, between −28.3 and −21.9‰. The oil was depleted in carbon-13 compared to the kernel with approx. 0.6‰, while the residue was enriched in carbon-13 compared to the kernel with approx. 1.9‰ (Table 2). 

In photosynthesis, C3 plants transform the carbon dioxide (*δ*^13^C = −8‰) in carbohydrates which will be further used to form other classes of compounds, including proteins and fatty acids, processes that will take place as a general rule with a 1‰ depletion in carbon-13 for proteins and 6‰ for lipids, since the ^12^C isotope is favored for the reactions that take place [31]. The study done by Guo et al. [26] went in even more details and showed the differences between the *δ*^13^C of individual fatty acids, which decreased slightly (0.7‰) in the elongation of C_18:0_ from C_16:0_, increased significantly (1.3‰) in the first desaturation (from C_18:0_ to C_18:1_) and kept stable in the further desaturation from C_18:1_ to C_18:2_.

The statistical analysis of the data (both isotopic and compositional) showed that the *δ*^13^C of the kernel, oil, and residue were important in the geographical discrimination (with *p*-values < 0.0001), *δ*^13^C of the residue (containing mostly the proteins and carbohydrates of the walnut) was important in differentiating the year of harvest (*p*-value = 0.004), while no carbon-13 fingerprint was important in discriminating the walnuts according to composition. These correlations indicate an influence of the location and year of harvest on the carbon isotopic composition of the walnuts. It can be mentioned that if the discrimination is done between cultivars from a restricted area, the isotopic composition is influenced by the biological parameters (metabolism) rather than the climatic factors, though no strong correlations were found between the isotopic fingerprint and composition of the walnuts.

The mean carbon-13 values of the different walnut groups are also presented in Table 2. The samples from 2016 were slightly more enriched in carbon-13 than the ones produced in 2015, with differences of about 0.4, 0.5 and 1.1‰ for the kernels, oil and residue. The trend is opposite than in other findings in which the isotopic fingerprint is enriched in the warmer years, though the two samples from Greece (Tripoli area) showed a more enriched isotopic composition for the oil and residue in the warmer year (2015).

**Figure 2:**
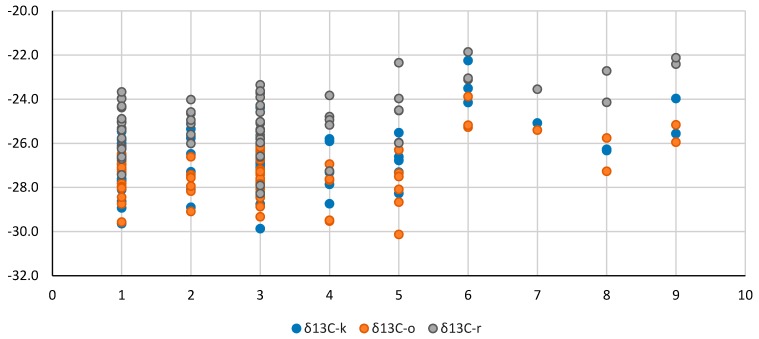
Carbon-13 composition of the kernel, oil and residue taking into account the geographical origin of the cultivars (1 = SCDP-USA cultivars, 2 = SCDP-FR cultivars, 3 = SCDP-RO cultivars, 4 = SCDP-RO selections, 5 = SCDP-RO hybrids, 6 = RO-VL, 7 = RO-DB, 8 = GR, 9 = PRC).

Concerning the geographical origin of the cultivars, a trend of isotope enrichment was observed, from the walnuts produced in the Fruit Growing Research Station (SCDP) Vâlcea (kernel of −27‰, oil of −28‰ and residue of −25‰), to the ones produced in Greece, followed by the red kernel walnut from Romania–Damboviţa county and walnuts from China, ending with the three walnut selections from Vâlcea county that registered the highest *δ*^13^C values (−23.3‰ for kernel, −24.8‰ for oil, and −22.7‰ for residue). The data are also presented as a distribution in Figure 2.

Using only the isotopic values, the walnuts can be differentiated according to the geographical origin, with the exception of the two samples from Greece which were classified in the RO–SCDP Vâlcea group, while the year of harvest discrimination gave only a 73.33% correct classification of the samples (11 out of 15). If the chemical composition is taken into account, groups C5 and C4 were the most enriched in carbon-13 (with the exception of C5 kernel), while groups C1, C2, and C3 were the most depleted.

### 2.3. Composition Discrimination

Nonspecific statistical analysis of the composition and isotopic data by agglomerative hierarchical clustering (AHC) classified the groups as presented in Table 3. The most important factors to differentiate these groups were the fat content, the energy of the oil, the oleic and linoleic concentrations, and the iodine value. 

Groups C1 and C2 were the most similar, with the next level of similarity clustering groups C1 and C2 with C3, followed by group C5, while group C4 had the most distinct composition. Groups C1–C3 had the most unsaturated composition (high polyunsaturated acids concentrations resulting in high IV values), with group C1 being distinct due to the high protein content and group C3 due to the higher SFA content; group C5 (formed of Hartley and Vina cultivars harvested in 2016) had walnuts with low fat and protein compositions, but high in SFA and linolenic acid. Group C4, where Adams 10, Lara, one Chinese cultivar (Jin Bo Feng no.1), and other five Romanian cultivars all harvested in 2016 were found, had a walnut composition high in fats with increased oleic acid concentrations.

Group C1 is comprised mainly from samples of 2015 harvest; groups C3–C5 are formed of walnuts harvested in 2016, while group C2 is a mixture of 2015 and 2016 samples. For the cultivar Wilson Franquette, the walnuts sampled in both years of harvest were classified in the same group, indicating a metabolism that is less influenced by the environmental conditions. For the other cultivars, however, the walnuts obtained in different years were classified in different groups, more or less related to each other due to the difference in composition given by the production year.

Concerning the Romanian walnut cultivars, selections and hybrids, the majority of them were classified in the high unsaturation groups (C1–C3), with the exception of five of them produced in 2016 which had a composition high in fats and oleic acid (group C4).

## 3. Materials and Methods

### 3.1. Plant Material and Collection Site

The main biological material analyzed in this study consisted of walnut kernels of fruits harvested from the germplasm collection of the Fruit Growing Research Station (SCDP) belonging to the University of Craiova and located in Râmnicu Vâlcea, Romania. The aim of SCDP is to improve the walnut assortment in Southwest Romania through selection of local walnut population and controlled hybridization. The walnut cultivar collection field is located north of Râmnicu Vâlcea city, at 45°8′22.35″N and 24°22′36.85″E. The climate of the area is temperate, of Cfb Köppen-Geiger type [32], the average annual temperature is 10.2 °C and rainfall 715 mm. The walnut collection was planted in 1997 on an alluvial soil with medium content of nitrogen, phosphorus, and potassium, and with a pH of 6.8. No irrigation was provided to the trees. Fruits were harvested at full maturity, in 2015 and/or 2016, from a number of 11 walnut representative cultivars originated from USA (seven from California, three from Oregon and one from Idaho), four cultivars from France, and 14 from Romania. Additionally, five advanced selections and six hybrids from the breeding program were sampled. Another set of samples was represented by walnuts grown in other locations than SCDP: Two cultivars from both Greece (Tripoli City area) and China (Taigu county and Taiyuan City from Shanxi Province), and the other four local selections from Romania (three from Vâlcea county and one with red kernel from Dâmboviţa county). A total of 64 samples were investigated, from which 15 were taken both in 2015 and 2016 (see Table 4).

### 3.2. Sample Preparation

After harvest, the in-shell walnuts were naturally dried. Nuts were cracked, and kernels were extracted from each cultivar sample. The mean sample consisted of 30 g of healthy kernels selected to be representative for the lot, grounded to a fine homogenous mix. From this, 1 g was dried by lyophilization and C/H/N composition and *δ*^13^C-IRMS were performed. Another 5 g of mean grounded walnut kernels were used to extract oil for 16 h with 100 mL of petroleum ether using a Buchi B-811 Soxhlet extractor (Büchi Labortechnik AG, Flawil, Switzerland). The oil resulted after the removal of the solvent and filtration represented the fat content of the kernels and its composition was determined–fatty acids content by NMR, C/H/N and *δ*^13^C-IRMS. The dried residue after oil extraction (consisting mainly of the proteins and carbohydrates of the walnuts) was also analyzed for the C/H/N composition and *δ*^13^C-IRMS.

### 3.3. C/H/N Composition

The elemental composition (C/H/N) of the walnut, oil, and residue was determined in triplicate using a Flash2000 Thermo (Thermo Fisher Scientific, Leicestershire, UK) with a TCD detector (Thermo Fisher Scientific, UK) [33]. Analysis conditions: Chromatographic columns—(i) molecular sieve 5A for separating flue gases after combustion, gas type CO, and (ii) PoraPLOT Q (30 cm, 20 μm, 65 °C) for separating flue gases after combustion, gas type NO_2_, CO_2_, H_2_O, SO_2,_ furnace temperature: 950 °C, TCD detector: 1000 mA, measuring time—12 min, sample mass 2.5 mg. Linear regression analysis, based on seven points created using certified reference material, traceable at ISO GUIDE 34/17025, was used to generate the calibration equation for each element. The condition of a correct measurement was to draw a calibration curve with an RSD < 0.5% and the correlation coefficients r^2^ > 0.99. To verify the conformity of the results or if the calibration curve did not suffer changes, before every sample injection, a quality control (QC) gas mixture, rich in permanently gases and hydrocarbons, was used. The measured values were used to calculate the energy values for walnut, oil and residue, and the proximate composition, crude protein (N × 5.30) (AOAC 950.48) and carbohydrate content.

### 3.4. NMR Analysis

The walnut oil samples for the NMR analysis were prepared in duplicate by dissolving 70 mg of oil in 700 μL of CDCl3 (99.96% at.) with TMS (NMR grade) as internal standard. The reagents were purchased from Aldrich (St. Louis, MO, USA) and, respectively, Sigma-Aldrich (Darmstadt, Germany). All NMR experiments were recorded at 300 K using a Bruker AvanceIII400 spectrometer (Bruker France SAS, Wissembourg, France) operating at 9.4 T, equipped with a 5 mm BBO probe, observing ^1^H at 400.2 MHz and ^13^C at 100.6 MHz. ^1^H-NMR spectra were acquired using spectral width of 8224 Hz; 65536 data points; pulse width of 10.5 μs; relaxation delay of 1.0 s; acquisition time of 4 s and 16 scans. To verify the spectra quality, the solvent peak was examined on the ^1^H spectrum for each sample. Since the same quantity of deuterated chloroform was always added for the NMR analysis, the resolution of the peak at 7.3 ppm was used to attest the correct adjustment of the shims. Spectra were processed by applying an exponential line broadening of 0.3 Hz for sensitivity enhancement before Fourier transforms and were accurately phased and baseline adjusted. The determination of the fatty acids composition of the walnut oil was done according to Guillén and Ruiz [34].

#### 3.5. *δ*^13^C Measurement

The overall carbon-13 composition of the walnut kernel, oil, and residue was obtained using a Thermo DeltaVPlus Spectrometer (Thermo Fisher Scientific, Bremen, Germany) coupled with an 1112 Elemental Analyser equipped with an autosampler for solids. The isotopic composition is expressed as *δ*-values (‰), which refers the isotope ratio of the sample (S) to that of the international reference PDB:

$$\delta^{13}C=1000\cdot \left[\frac{{\left({}^{13}C/{}^{12}C\right)}_{S}-{\left({}^{13}C/{}^{12}C\right)}_{\mathrm{PDB}}}{{\left({}^{13}C/{}^{12}C\right)}_{\mathrm{PDB}}}\right]$$

The *δ*^13^C isotopic values were calibrated against an international reference material, sugar IAEA-CH-6 (IAEA—International Atomic Energy Agency, Vienna, Austria), and three other laboratory standards, namely, L-Alanine IA-R041 (Iso-Analytical Laboratory Standard), Beet Sugar IA-R005, and Cane Sugar IA-R006.

During measurements, a reference was injected at regular intervals as a working standard to check the accuracy of the results. Each sample was analyzed in triplicate and the results were validated if the difference was below 0.4‰.

### 3.6. Data Analysis

The data were evaluated by statistical methods. Descriptive statistical analysis (mean, standard deviation, etc.), agglomerative hierarchical clustering (AHC), and discriminant analysis (DA) were performed using commercial software packages as Microsoft Excel 2013 (Microsoft, Redmond, WA, USA) and XLSTAT Addinsoft 2014.5.03 version (Addinsoft Inc, Long Island, NY, USA).

## 4. Conclusions

The determined parameters (fat, protein, nutritional value, fatty acids profile by ^1^H-NMR, and carbon-13 isotopic composition) for the walnuts and walnut oil were used to differentiate with certain degrees of success the cultivars grown in the same location or different locations and to show the composition differences for the same cultivar in different years of production. The study allowed the comparison of the Romanian cultivars, selections and hybrids with the internationally available ones. The data also showed that in order to enhance the discrimination of walnut and walnut oil samples a wider range of parameters is needed, for example to couple the isotopic fingerprint (of bulk, fractions, or individual compounds) with the composition data or vice versa.

## Figures and Tables

**Table 1:** Composition of the walnut kernel, oil, and residue.

Geographical Origin/Composition	All Samples (*n* = 64)	Germplasm Collection of Fruit Growing Research Station (SCDP) Vâlcea, Grown to RO-VL1							RO-VL2 Local Selections (*n* = 3)	RO-DB Red Kernel (*n* = 1)	GR (*n* = 2)		PRC (*n* = 2)	
		USA Cultivars (*n* = 18)	FR Cultivars (*n* = 7)	RO Cultivars (*n* = 20)	RO Selections (*n* = 5)	RO Hybrids (*n* = 6)	Samples Collected in Both 2015 and 2016 (*n* = 30)							
							2015	2016			GR-1	GR-2	PRC-1	PRC-2
kernel														
Total fat-k (%)	69.0 ± 7.6	66.6 ± 7.1	73.6 ± 6.1	71.0 ± 9.3	66.3 ± 2.3	66.5 ± 3.9	66.4 ± 4.0	73.4 ± 10.7	66.7 ± 4.0	79.6	72.0	60.6	79.4	61.1
Protein-k (%)	20.3 ± 6.4	19.8 ± 7.9	19.7 ± 5.9	20.1 ± 6.3	22.8 ± 4.9	26.0 ± 2.2	26.2 ± 4.6	13.3 ± 2.2	18.1 ± 4.5	16.7	12.9	17.4	14.6	15.6
Carbohydrates-k (%)	10.7 ± 8.0	13.5 ± 9.8	6.7 ± 1.8	8.9 ± 8.3	10.8 ± 5.9	7.5 ± 5.5	7.3 ± 5.7	10.2 ± 0.9	15.2 ± 0.9	3.7	15.1	22.1	6.0	23.2
Energy-k (kcal/100 g)	700 ± 22	703 ± 24	699 ± 19	699 ± 22	693 ± 9	702 ± 33	696 ± 21	709 ± 21	693 ± 18	713	730	698	704	711
oil														
SFA-o (%molar)	10.6 ± 0.9	10.2 ± 0.8	10.9 ± 0.7	10.7 ± 1.0	10.5 ± 0.7	10.8 ± 0.8	10.7 ± 0.7	10.2 ± 0.9	11.1 ± 0.7	13.4	11.0	12.3	8.9	9.3
Oleic-o (%molar)	22.7 ± 5.2	23.6 ± 5.5	21.3 ± 3.4	23.1 ± 4.2	24.7 ± 5.6	18.8 ± 3.5	19.9 ± 3.2	26.1 ± 5.2	16.1 ± 2.4	20.9	19.9	20.2	38.5	31.2
Linoleic-o (%molar)	55.6 ± 4.6	55.2 ± 5.0	56.3 ± 2.1	55.1 ± 4.0	54.8 ± 3.9	59.2 ± 5.3	58.0 ± 3.3	52.7 ± 4.4	60.5 ± 1.5	54.1	55.2	55.7	43.4	50.6
Linolenic-o (%molar)	11.1 ± 1.6	11.1 ± 1.6	11.5 ± 1.7	11.0 ± 1.4	10.1 ± 2.0	11.2 ± 2.1	11.4 ± 2.0	11.0 ± 1.0	12.4 ± 0.4	11.6	14.0	11.8	9.3	8.9
IV-o	139 ± 4	138 ± 4	140 ± 4	138 ± 4	137 ± 6	142 ± 2	141 ± 4	136 ± 4	144 ± 1.3	134	141	138	126	131
PUFA (%molar)	66.7 ± 4.8	66.2 ± 5.0	67.8 ± 3.3	66.2 ± 4.0	64.9 ± 5.2	70.4 ± 3.4	69.4 ± 3.3	63.7 ± 4.7	72.9 ± 1.7	65.7	69.2	67.5	52.6	59.5
UFA/SFA	8.49 ± 0.83	8.9 ± 0.8	8.2 ± 0.6	8.38 ± 0.78	8.58 ± 0.62	8.30 ± 0.66	8.43 ± 0.63	8.87 ± 0.96	8.07 ± 0.6	6.46	8.13	7.12	10.2	9.8
PUFA/SFA	6.32 ± 0.55	6.5 ± 0.5	6.3 ± 0.5	6.21 ± 0.62	6.20 ± 0.39	6.55 ± 0.57	6.55 ± 0.57	6.26 ± 0.51	6.61 ± 0.3	4.90	6.31	5.48	5.92	6.43
Energy-o (kcal/100 g)	874 ± 69	900 ± 56	848 ± 60	854 ± 71	892 ± 11	886 ± 11	886 ± 18	857 ± 104	887 ± 9	816	903	903	744	902
residue														
Protein-r (%)	41.3 ± 6.7	39.8 ± 6.8	41.7 ± 2.6	40.2 ± 7.6	43.7 ± 5.3	40.7 ± 6.0	42.5 ± 7.9	38.7 ± 6.0	52.3 ± 6.2	34.7	39.2	44.1	45.3	46.2
Energy-r (kcal/100 g)	303 ± 17	307 ± 23	293 ± 6	303 ± 18	300 ± 8	310 ± 16	312 ± 23	295 ± 11	304 ± 7	312	286	289	297	300

k—kernel, o—oil, r—residue.

**Table 2:** Stable carbon composition (mean values) of the investigated walnut samples.

		*δ*^13^C-Kernel (‰)	*δ*^13^C-Oil (‰)	*δ*^13^C-Residue (‰)
All samples (*n* = 64)		−26.8 ± 1.5	−27.4 ± 1.2	−24.9 ± 1.4
Samples collected in both years	2015 (*n* = 15)	−27.3 ± 1.4	−27.6 ± 0.8	−25.6 ± 1.3
	2016 (*n* = 15)	−26.9 ± 1.4	−27.1 ± 0.7	−24.5 ± 0.7
Samples growth to SCDP	USA cultivars (*n* = 18)	−27.4 ± 1.2	−27.5 ± 0.8	−25.3 ± 1.1
	RO selections (*n* = 5)	−27.1 ± 1.3	−28.3 ± 1.2	−25.2 ± 1.3
	RO cultivars (*n* = 20)	−27.0 ± 1.2	−27.4 ± 0.9	−25.2 ± 1.4
	RO hybrids (*n* = 6)	−26.9 ± 0.9	−28.0 ± 1.3	−24.5 ± 1.4
	FR cultivars (*n* = 7)	−26.8 ± 1.4	−27.8 ± 0.8	−25.0 ± 0.7
Samples from Greece	GR-2 2016	−26.3	−27.3	−24.1
	GR-1 2015	−26.3	−25.8	−22.7
Samples from China	PRC-2 (Zhong Lin no.1)	−25.6	−25.2	−22.1
	PRC-1 (Jin Bo Feng no.1)	−24.0	−26.0	−22.4
Samples from Romania (other than SCDP)	RO-DB red kernel selection (*n* = 1)	−25.1	−25.4	−23.6
	RO-VL (*n* = 3)	−23.3 ± 1.0	−24.8 ± 0.8	−22.7 ± 0.7
Samples classified by groups	C1 (*n* = 23)	−27.0 ± 1.7	−27.6 ± 1.3	−25.4 ± 1.6
	C2 (*n* = 25)	−26.6 ± 1.2	−27.4 ± 1.2	−24.7 ± 1.3
	C3 (*n* = 6)	−26.8 ± 1.4	−27.4 ± 1.1	−24.8 ± 1.1
	C4 (*n* = 8)	−26.3 ± 1.5	−26.8 ± 0.7	−24.1 ± 1.0
	C5 (*n* = 2)	−28.2	−26.5	−24.0

**Table 3:** Classification of the walnuts according to composition.

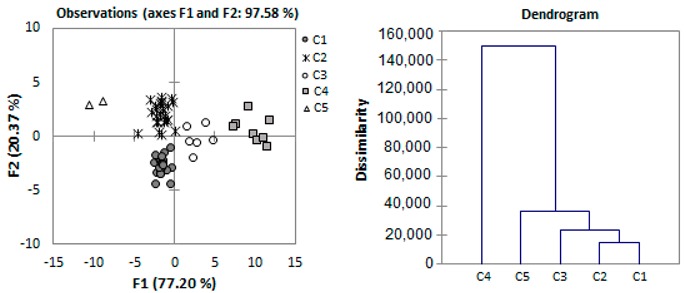				
Group Labels	Group Components			
	SCDP–Cultivars	SCDP–Romanian Selections	SCDP–Romanian Hybrids	Other Geographical Origin
C1 (*n* = 23)	USA: Chase D9-2016, Hartley 2015, Pedro 2015, Tehama 2015, Vina 2015 FR: Fernette 2015, Fernor 2015, Lara 2015, RO: Argesan 2015, Germisara 2015, Muscelean 2015, Orastie 2015, Roxana 2015, Sibisel 44-2015, Sibisel Precoce 2015, Valcris-2015	Munteanu 2015, VL206S 2015, Vladesti 2015	HC 02-2015, UC 4/12-2015, T 2/3-2015	Fumureni 2-2016 (RO-VL2)
	C1 compositional characteristics: high protein, linoleic, linolenic, IV			
C2 (*n* = 25)	USA: Adams 10-2015, Chase D9-2015, Howe 2016, Idaho 2016, Payne 2016, Pedro 2016, Serr 2016, Wilson Franquette 2015/2016 RO: Germisara 2016, Mihaela 2015, Muscelean 2016, Timval 2015, Valcor 2016, Velnita 2015	Ignat Tudor 2015, VL 301B-2015	C2-2015, HC3390-2015, UC 3/1-2015	Brezoi-2015 (RO-VL2), Fumureni 1-2016 (RO-VL2), GR 1-2015, GR 2-2016, PRC 2-2016 (Zhong Lin no.1 cultivar)
	C2 compositional characteristics: high linoleic, linolenic, IV			
C3 (*n* = 6)	USA: Payne 2015 FR: Ferjean 2016, Fernette 2016, Fernor 2016 RO: Jupanesti 2015	-	-	Targoviste 1-red kernel 2016 (RO-DB)
	C3 compositional characteristics: high SFA, linoleic, linolenic, IV			
C4 (*n* = 8)	USA: Adams 10-2016, FR: Lara 2016 RO: Roxana 2016, Sibisel 44-2016, Timval 2016, Unival 2016, Valmit 2016	*-*	*-*	PRC 1-2016 (Jin Bo Feng no.1 cultivar)
	C4 compositional characteristics: high fat, oleic, UFA/ SFA; low linoleic, linolenic, IV			
C5 (*n* = 2)	USA: Hartley 2016, Vina 2016	-	-	-
	C5 compositional characteristics: low fat, proteins; high SFA, linolenic			

**Table 4:** Description of walnut samples collection.

Geographical Origin of the Cultivar	Description	Name	Genetic Origin	Harvest Year
Fruit Growing Location-Research Station (RO-VL1), Romania				
USA (Oregon)	Cultivar	Adams 10	Open pollinated seedling	2015 and 2016
	Cultivar	Chase D9	Open pollinated seedling	2015 and 2016
	Cultivar	Howe	Chance seedling	2016
USA (California)	Cultivar	Tehama	‘Waterloo’ × ‘Payne’	2015
	Cultivar	Hartley	Open pollinated seedling	2015 and 2016
	Cultivar	Payne	Chance seedling	2015 and 2016
	Cultivar	Pedro	‘Conway Mayette’ × ‘Payne’	2015 and 2016
	Cultivar	Vina	‘Franquette’ × ‘Payne’	2015 and 2016
	Cultivar	Wilson Franquette	Selection of ‘Franquette’	2015 and 2016
	Cultivar	Serr	‘Payne’ × PI 159568	2016
USA (Idaho)	Cultivar	Idaho	Selection from local populations	2016
France	Cultivar	Fernette	‘Franquette’ × ‘Lara’	2015 and 2016
	Cultivar	Fernor	‘Franquette’ × ‘Lara’	2015 and 2016
	Cultivar	Lara	Chance seedling of ‘Payne’	2015 and 2016
	Cultivar	Ferjean	Grosvert’ × ‘Lara’	2016
Romania (Argeş)	Cultivar	Argesan	Selection from local populations	2015
	Cultivar	Jupâneşti	Selection from local populations	2015
	Cultivar	Mihaela	Selection from local populations	2015
	Selection	Ignat Tudor	Selection from local populations	2015
	Selection	Munteanu	Selection from local populations	2015
	Selection	Vladesti	Selection from local populations	2015
	Cultivar	Muscelean	Selection from local populations	2015 and 2016
	Cultivar	Roxana	Selection from local populations	2015 and 2016
Romania (Hunedoara)	Cultivar	Sibisel Precoce	Selection from local populations	2015
	Cultivar	Orastie	Selection from local populations	2015
	Cultivar	Germisara	Selection from local populations	2015 and 2016
	Cultivar	Sibişel 44	Selection from local populations	2015 and 2016
Romania (Vâlcea)	Cultivar	Valcris (syn. VL202 PO)	Selection from local populations	2015
	Selection	VL 206 S	Selection from local populations	2015
	Cultivar	Timval (syn. VL 54 B)	Selection from local populations	2015 and 2016
	Cultivar	Unival	Selection from local populations	2016
	Cultivar	Valcor	Selection from local populations	2016
	Cultivar	Valmit	Selection from local populations	2016
Romania (Craiova)	Hybrid	C2	Open pollinated seedling of ‘Ideal’	2015
	Hybrid	HC 02	Open pollinated seedling of ‘Ideal’	2015
	Hybrid	HC 3390	Open pollinated seedling of ‘Ideal’	2015
	Hybrid	T2/3	Open pollinated seedling of ‘Ideal’	2015
	Hybrid	UC 3/1	Open pollinated seedling of ‘Ideal’	2015
	Hybrid	UC 4/12	Open pollinated seedling of ‘Ideal’	2015
Romania (Bucureşti)	Selection	VL 301 B	Selection from local populations	2015
Romania (Iaşi)	Cultivar	Velniţa	Selection from local populations	2015
Fruit Growing Location-Vâlcea county (RO-VL2), Romania				
Romania (Vâlcea)	Selection	Brezoi 1	Selection from local populations	2016
	Selection	Fumureni 1	Selection from local populations	2016
	Selection	Fumureni 2	Selection from local populations	2016
Fruit Growing Location-Dâmboviţa county (RO-DB), Romania				
Romania (Dâmboviţa)	Selection	Târgovişte 1-red kernel	Selection from local populations	2016
Fruit Growing Location-Tripoli (GR), Greece				
Greece (Tripoli)	Cultivar	Nut sample 1	Franquette’ × ‘Hartley’ x ‘Chandler’ × ‘Meylannaise’	2015
Greece (Tripoli)	Cultivar	Nut sample 2		2016
Fruit Growing Location-PRC				
China (Fruit Growing Institute in Taigu-Shanxi)	Cultivar	Jin Bo Feng no.1	Unknown	2016
China (Taiyuan market, Shanxi)	Cultivar	Zhong Lin no.1	Unknown	2016
